# Age-related changes in platelet function are more profound in women than in men

**DOI:** 10.1038/srep12235

**Published:** 2015-07-16

**Authors:** Jonathan Cowman, Eimear Dunne, Irene Oglesby, Barry Byrne, Adam Ralph, Bruno Voisin, Sieglinde Müllers, Antonio J. Ricco, Dermot Kenny

**Affiliations:** 1 Molecular and Cellular Therapeutics, Royal College of Surgeons in Ireland, Dublin, Ireland; 2 Biomedical Diagnostics Institute, Dublin City University, Dublin, Ireland; 3 Irish Centre for High-end Computing, National University Ireland, Galway, Ireland; 4 Royal College of Surgeons in Ireland, Rotunda Hospital, Dublin, Ireland

## Abstract

Age is a risk factor for cardiovascular disease (CVD), however the effect of age on platelet function remains unclear. Ideally, platelet function should be assayed under flow and shear conditions that occur *in vivo*. Our study aimed to characterise the effect of age on platelet translocation behaviour using a novel flow-based assay that measures platelet function in less than 200 μl of blood under conditions of arterial shear. Blood from males (n = 53) and females (n = 56), ranging in age from 19–82 and 21–70 respectively were perfused through custom-made parallel plate flow chambers coated with immobilised human von Willebrand Factor (VWF) under arterial shear (1,500s^−1^). Platelet translocation behaviour on VWF was recorded by digital-image microscopy and analysed. The study showed that aging resulted in a significant decrease in the number of platelet tracks, translocating platelets and unstable platelet interactions with VWF. These age related changes in platelet function were more profound in women than in men indicating that age and gender significantly impacts on platelet interactions with VWF.

Age affects most cellular processes but the effect of age on platelet function is unclear. Most studies to date have used non-physiological approaches such as light transmission aggregometry (LTA) or flow cytometry to investigate age-related changes in platelet function^1^,^2^,^3^,^4^. These studies have reported that aging results in significant increases in the release of plasma β-thromboglobulin (βTG), plasma platelet factor 4 (PF4), phosphoinositide turnover, platelet aggregation and plasma fibrinogen^5^,^6^,^7^.

Ideally assays of platelet function should replicate the flow and shear environment that platelets experience *in vivo*. The platelet function analyser, the PFA-100 assesses platelet function under high-shear conditions. The device measures the time taken for platelets to occlude an aperture that is coated with either collagen and epinephrine (CEPI) or collagen and ADP (CADP). The results of the PFA-100 are reported as closure time (CT)^8^. A number of studies have used the PFA-100 to assess age-related changes in platelet function. However, the results of these studies are inconsistent. For example, a PFA-100 study of healthy Koreans (n = 78 males and 42 females), identified that older Koreans (>40 years) had platelet CT’s that were significantly shorter than those observed in younger populations. The study also concluded that females had longer CT’s compared to males^9^. In contrast to this study in healthy Koreans, Sestito and colleagues investigated the effects of age and gender on platelet CTs in 62 apparently healthy individuals (n = 33 males and 29 females), 35 to 75 years of age. The results of that study demonstrated an absence of correlation between age, gender and platelet CT^10^.

During vascular damage, vascular matrix proteins are exposed to the flowing blood plasma. Von Willebrand Factor (VWF) by binding to the exposed subendothelial matrix, tethers circulating platelets in flow via the platelet GPIbα receptor^11^. These initial interactions between VWF and the platelet are short-lived, resulting in platelet translocation (stop/start motion of the platelet) as GPIbα-VWF bonds are formed and broken^12^. The net result is activation and adhesion of the platelet to the exposed surface^13^. Our research group has developed a Dynamic Platelet Function Assay (DPFA), using novel parallel plate flow chambers coated with immobilised human VWF and custom-designed platelet tracking software^14^. This system measures platelet translocation behaviour on VWF.

In this study, we employed the above approach to investigate the effect of age on platelet function in over 100 healthy donors. Four novel parameters relating to the different stages of platelet interaction with VWF were measured.

## Results

### Evaluation of the effects of aging on platelet function in the DPFA

#### Platelet behaviour on VWF is significantly altered with aging

**Figure 1:**
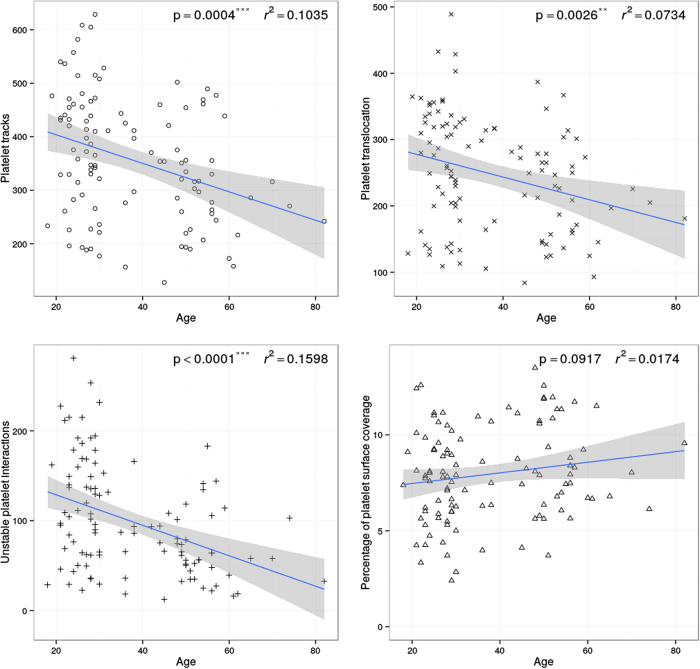
Platelet behaviour on VWF significantly changes with age. Blood taken from 109 healthy donors was perfused over VWF at arterial shear. Platelet translocation behaviour on VWF was measured. Age resulted in a significant change in the behaviour of platelets translocating on VWF.

A total of 109 subjects were studied. Aging resulted in a significant reduction in the number of platelet tracks (slope ***p = 0.0004, r^2^ = 0.1035), translocating platelets (slope **p = 0.0026, r^2^ = 0.0734) and numbers of unstable platelet interactions (slope ***p < 0.0001, r^2^ = 0.1598) with VWF. Aging resulted in a trend towards an increased percentage of platelet surface coverage but this was not statistically significant (slope p = 0.0917, r^2^ = 0.0134) (Fig. 1).

### Evaluation of the effects of gender and age on platelet function in the DPFA

#### Changes in platelet function associated with age are more profound for women than in men

Having demonstrated that platelet translocation behaviour was significantly altered with age, the effect of age on platelet function in men (n = 53) and women (n = 56) was investigated.

**Figure 2:**
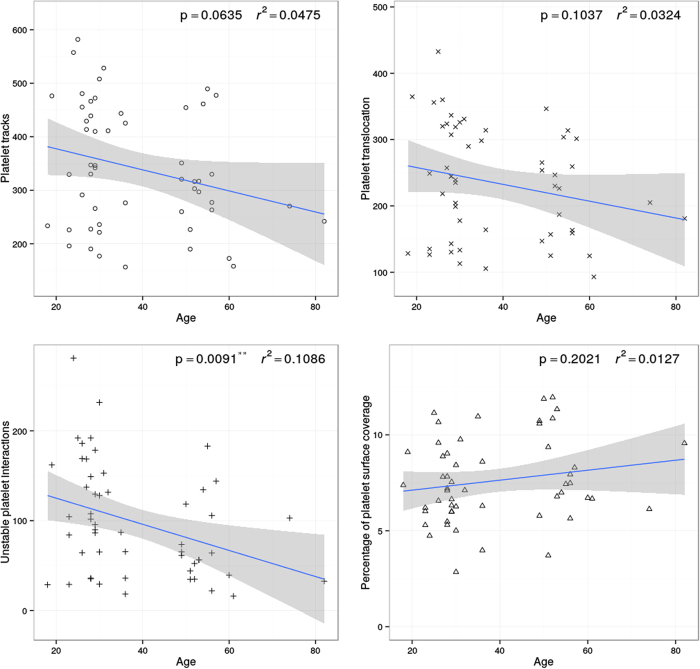
Platelet behaviour on VWF in males is altered with age. Blood taken from 53 males was perfused over VWF at arterial shear. Platelet translocation behaviour on VWF was measured. Aging in males resulted in a significant reduction in the numbers of unstable platelet interactions with VWF.

Aging in men resulted in a trend towards decreased numbers of platelet tracks but this was found not be statistically significant (slope p = 0.0635, r^2^ = 0.0475). No significant change was observed in the number of translocating platelets (slope p = 0.1037, r^2^ = 0.0324). However, aging in men resulted in a significant decrease in the number of unstable platelet interactions (slope **p = 0.0091, r^2^ = 0.1086) with VWF. No significant changes were observed with age in the percentage of platelet surface coverage (slope p = 0.2021, r^2^ = 0.0127) in men (Fig. 2).

**Figure 3:**
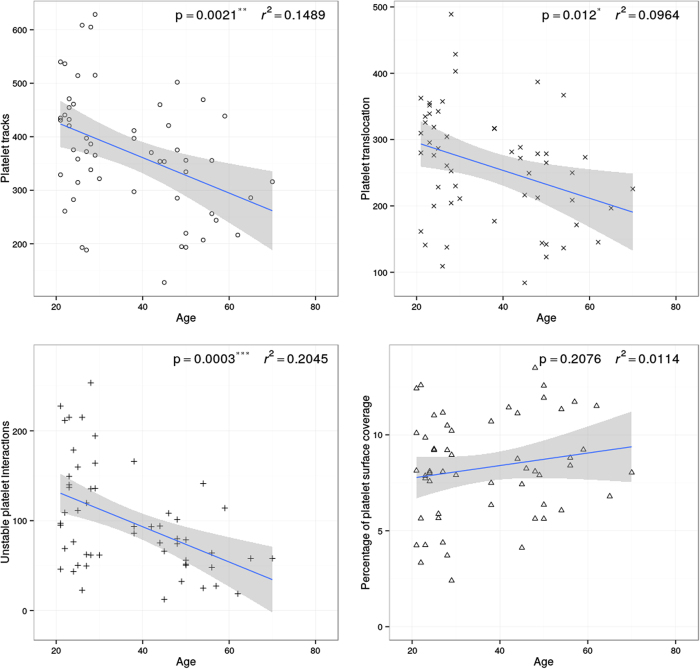
Platelet behaviour on VWF in females is altered with age. Blood taken from 56 females was perfused over VWF at arterial shear. Platelet translocation behaviour on VWF was measured. Aging in females resulted in a significant change in the behaviour of platelets translocating on VWF.

Aging in females resulted in a significant reduction in the number of platelet tracks (slope **p = 0.0021, r^2^ = 0.1489), translocating platelets (slope *p < 0.0120, r^2^ = 0.0964) and numbers of unstable platelet interactions (slope ***p = 0.0003, r^2^ = 0.2045) with VWF. There was no significant difference in the percentage of platelet surface coverage (slope p = 0.2076, r^2^ = 0.0114) in females with age (Fig. 3).

## Discussion

The results of this study demonstrate that age has a significant effect on platelet translocation behaviour on VWF. The effects of age on platelet function are more profound in women than in men.

Previous studies have reported that platelet activation increases with age. For example, Bastyr and colleagues demonstrated increased platelet phospholipid content, suggesting increases in transmembrane signalling with age^6^. It has also been shown that age is associated with an increase in platelet aggregability^3^. Reilly and FitzGerald showed that bleeding times were significantly reduced in older subjects^15^. Our study used a novel assay that assesses platelet function in real time and characterised platelet behavior in a cohort of healthy individuals. Overall, our results demonstrate that age causes a significant a change in the behaviour of platelets translocating on VWF. This is readily apparent where age is associated with significant decreases in numbers of platelet tracks, platelet translocation and numbers of unstable platelet interactions with VWF. There was a trend towards increased percentages of platelet surface coverage but this was found not to be statistically significant. It is unlikely that these changes in platelet function associated with aging are a result of changes in plasma levels of VWF. Although, we did not measure plasma levels of VWF in this study, it was previously demonstrated by Wu and co-workers that plasma levels of VWF have no significant effect on platelet adhesion to glass surfaces coated with immobilised VWF^16^. Our study has identified that aging results in a change in the behaviour of platelets interacting with VWF.

The results demonstrate that age associated changes in platelet function on VWF are more pronounced in women than in men. This may be related to the potential influence of sex hormones such as testosterone and estrogen. Previous studies of platelet function have suggested that testosterone and estrogen can have a regulatory role in platelet function^17^,^18^,^19^. Testosterone has been shown to significantly enhance platelet thromboxane A_2_ receptor density and heighten platelet aggregation responses to arachidonic acid^18^. Platelets express the estrogen receptor β^20^,^21^ and deletion of the estrogen receptor β in aged female mice results in the amplification in the thrombogenicity of platelets^22^. In our study, the more pronounced change in platelet translocation behaviour on VWF associated with age in females when compared to that of males may be reflective of a reduction in estrogen levels experienced in women rather than the gradual decline of testosterone levels in men associated with aging. This is recommended as an area for future research.

There is a paucity of studies on the influence of age and gender on platelet adhesion to vascular proteins. Significantly this study demonstrates, for the first time, that age and gender significantly impacts on platelet adhesion to VWF under arterial shear conditions. In particular, we demonstrated decreases in platelet tracks, platelet translocation and unstable platelet interactions with age. These differences in platelet function associated with aging were more profound with age in females compared with that of males. Although, we did not formally evaluate menopausal status in these women, these more profound changes in platelet function in females could be due of a reduction in estrogen levels experienced in women rather than the gradual decline of testosterone levels in men. This novel DPFA with its multi-parameter analysis of platelet translocation behaviour on VWF is capable of detecting subtle changes in platelet function. These age related alterations in platelet function may contribute to the increased incidence of thrombotic events seen with aging.

## Methods

### Ethics statement

Ethical approval for the study was obtained from the Medical Research Ethics Committee of the Royal College of Surgeons in Ireland. All participants were informed of the nature of the study and written consent was obtained from all donors prior to recruitment. All blood samples were collected in accordance with the declaration of Helsinki.

### Study participants

A total of 109 participants were included in the study (Table 1). The inclusion criteria were: healthy volunteers with no previous history of any disease or illness and taking no prescribed medication such as statins, blood pressure tablets, aspirin etc or medications such ibuprofen, 12 days prior to blood draw. Those volunteers that did not meet these inclusion criteria’s for the study were excluded.

### Preparation of blood

Venous blood was drawn from the antecubital vein using a 19-gauge butterfly needle connected to a sterile polypropylene syringe. Blood was drawn into 3.2% (w/v) trisodium citrate anticoagulant (1:9 volume of citrate to blood, final citrate concentration of 0.32%). Blood samples were kept at room temperature with gentle rocking and used within 1 hour of phlebotomy. Whole blood cell counts were recorded for each donor, using a Sysmex-KX21N haematology analyser (Sysmex Corp., Kobe, Japan).

### Preparation of parallel plate flow chambers and blood samples

Whole blood flow assays were performed using custom-designed parallel plate flow chambers originally described by Kent *et al.*^14^ but with some recent modifications to allow for a reduction in the amount blood required. In brief, pre-assembled, single-use microfluidic chambers were custom-designed to consist of a 25 × 55 mm polymethyl methacrylate (PMMA) top plate (Ensinger Plastics, UK) fitted with inbuilt 1/16-inch polypropylene inlet and outlet connectors, a polyester gasket defining the flow path and coated on both sides with acrylic adhesive (Adhesives Research, Limerick, Ireland), and a microscope coverslip (24 × 50 mm VWR Germany) were used. The double-sided adhesive gasket was used to seal the assembled chamber and provide a uniform flow path 50 μm deep, 2 mm wide, and 35 mm long. The channel of the parallel plate flow chamber was coated with 100 μg/mL of VWF (courtesy of Robert Montgomery, Blood research Institute, Milwaukee, WI, USA) that was diluted in phosphate buffered saline (PBS) buffer overnight at 4 °C. The channel was then washed 3 times with PBS, blocked with 1% (w/v) BSA at 1 hour at room temperature, followed by final 3 washes with PBS buffer prior to blood perfusion. 200 μL of blood was aliquoted into an eppendorf and incubated with 1 μM 3,3′-dihexyloxacarbocyanine iodide (DiOC_6_) lipophilic fluorescent dye (Invitrogen, Carlsbad, CA, USA) for 10 minutes at 37 °C.

### Perfusion of whole blood and image acquisition in parallel plate flow chamber

The parallel plate flow chambers were mounted on an inverted microscope (Zeiss Axiovert-200 epi-flouresence). Blood was drawn through biocompatible platinum-cured silicone tubing (Nalgene, 1/16 in internal diameter, Thermo Fisher Scientific, Denmark) and then perfused through the flow chamber, across the VWF-coated surface using a NEMESYS syringe pump (Cetoni GmbH, Korbussen, Germany). A flow rate (Q) of 75 μL/min was used corresponding to an arterial shear rate (γ) of 1,500 s^−1^ and a shear stress (τ) of 6 Pa (6 N/m^2^) at the wall of the surface where the platelets are interacting. Images were captured using a vacuum-cooled (−80 °C) digital EM-CCD camera (iXON EM+, Andor Technology, Belfast, Ireland) connected to MetaMorph (version 7.7, Molecular Devices Ltd., UK) illuminated with an Osram 103-W mercury light source and a fluorescein isothiocyanate (FITC) filter set providing excitation and emission at 490 nm and 528 nm, respectively (Chroma Technology Corp, Vermont, USA). Images were acquired at 30 frames per second (fps) for 500 frames using a 63x objective (512 × 512 pixel, 128.768 × 128.768 μm) field of view for visualisation of platelet interactions with the VWF in real time.

### Image analysis

The acquired images were analysed using a recently described platelet tracking software with some minor modifications^14^. In brief, the platelet algorithm accurately identifies individual platelets in each frame of an image sequence of 500 frames (corresponding to the first 16.7 seconds of image acquisition). The software using an Image Processing Toolkit in MatLab (version 7.12.0. (R2011a), Natick, Massachusetts: The MathWorks Inc, 2011) detects the size and (x, y) centroid position of each of the platelets in the imaged area against a continuously changing background. Platelet tracks are constructed as each individual platelet’s movement is tracked from one frame to the next. A weighted distance matrix is generated between the platelet track’s current position and other platelets on the frame that gives preference to platelet movement in the direction of flow over cross-stream movement. Each track is extended by assigning the original platelet to a platelet positioned in the next frame, using a set of rules to the weighted distance matrix. Each platelet in the image sequence is linked using a list of tracks with (x,y) positions and area (A) that relate to each individual platelet’s movement. The result is a list of platelet tracks corresponding to the associated positions over time for each platelet in an image sequence. The assay determines the numbers of platelets that interact with VWF (platelet tracks), the number of platelets that translocate on the surface (platelet translocation), the number of platelets that interact but do not adhere to the surface (unstable platelet interactions) and finally the percentage of platelets that remain stuck to the VWF surface on the final frame (percentage of platelet surface coverage).

A platelet track is defined as a platelet that interacts with VWF. A translocating platelet can is defined as platelets that travel >1.5 the average radius of the platelet. Unstable platelet interactions are defined as platelets that interact with VWF for a period that is greater than 10 frames but less than 490 frames. The percentage of platelet surface coverage is defined as the percentage of platelet surface coverage on the final frame (500).

### Statistical analysis

In order to address the variability of the assay related to blood condition and experimental error, each donor’s blood was run in triplicate in the DPFA. Each cohort (Males or Females) a mean and standard deviation is determined for each measured parameter (e.g. platelet tracks, platelet translocation etc.). Any of the 3 runs found to be outside the mean ±2SD of their respective cohort (Males or Females) are considered outliers and are removed prior to statistical analysis. This process facilitated detection and removal of extreme outliers to determine a normal reference range. The models exploring direct correlation between age and the measured platelet parameters are simple linear regression (single explanatory variable), calculated and plotted with R.

## Additional Information

**How to cite this article**: Cowman, J. *et al.* Age-related changes in platelet function are more profound in women than in men. *Sci. Rep.* **5**, 12235; doi: 10.1038/srep12235 (2015).

## Figures and Tables

**Table 1:** Demographics of study populations.

**Demographics**	**All**	**Males**	**Females**	**p values**
**Number**	109	53	56	–
**Age (Range)**	18–82	18–82	21–70	ns
**Platelet count (x 10**^**3**^ **per μL)**	205 ± 53	192 ± 35	205 ± 45	ns
**Haematocrit (%)**	39 ± 3	39 ± 3	38 ± 4	ns
**Smokers (%)**	4%	6%	2%	–

A Kruskal-Wallis test with a Dunns post test was used to determine statistical differences between variables. p > 0.05 were considered non-significant.
